# Derivatization of Rosmarinic Acid Enhances its in vitro Antitumor, Antimicrobial and Antiprotozoal Properties

**DOI:** 10.3390/molecules24061078

**Published:** 2019-03-19

**Authors:** Silvia Bittner Fialová, Martin Kello, Matúš Čoma, Lívia Slobodníková, Eva Drobná, Ivana Holková, Mária Garajová, Martin Mrva, Vlastimil Zachar, Miloš Lukáč

**Affiliations:** 1 Department of Pharmacognosy and Botany, Faculty of Pharmacy, Comenius University in Bratislava, Odbojárov 10, 832 32 Bratislava, Slovakia; 2 Department of Pharmacology, Faculty of Medicine, P. J. Šafárik University, Trieda SNP 1, 040 11 Košice, Slovakia; kellomartin@yahoo.com (M.K.); coma.matus@gmail.com (M.C.); 3 Institute of Microbiology, Faculty of Medicine, Comenius University in Bratislava, Sasinkova 4, 811 08 Bratislava, Slovakia; livia.slobodnikova@fmed.uniba.sk; 4 Department of Cell and Molecular Biology of Drugs, Faculty of Pharmacy, Comenius University in Bratislava, Kalinčiakov 8, 832 32 Bratislava, Slovakia; drobna@fpharm.uniba.sk (E.D.); holkova@fpharm.uniba.sk (I.H.); 5 Department of Zoology, Faculty of Natural Sciences, Comenius University in Bratislava, Mlynská dolina Ilkovičova 6, 842 15 Bratislava, Slovakia; martin.mrva@uniba.sk (M.G.); maria.garajova@uniba.sk (M.M.); 6 Department of Chemical Theory of Drugs, Faculty of Pharmacy, Comenius University in Bratislava, Kalinčiakov 8, 832 32 Bratislava, Slovakia; vlastimil.zachar@gmail.com

**Keywords:** rosmarinic acid, quaternary phosphonium salts, anticancer, antibacterial, MRSA, antifungal, anti-*Acanthamoeba* activity

## Abstract

On its own, rosmarinic acid possesses multiple biological activities such as anti-inflammatory, antimicrobial, cardioprotective and antitumor properties, and these are the consequence of its ROS scavenging and inhibitory effect on inflammation. In this study, two quaternary phosphonium salts of rosmarinic acid were prepared for the purpose of increasing its penetration into biological systems with the aim of improving its antimicrobial, antifungal, antiprotozoal and antitumor activity. The synthetized molecules, the triphenylphosphonium and tricyclohexylphosphonium salts of rosmarinic acid, exhibited significantly stronger inhibitory effects on the growth of HCT116 cells with IC_50_ values of 7.28 or 8.13 μM in comparison to the initial substance, rosmarinic acid (>300 μM). For the synthesized derivatives, we detected a greater than three-fold increase of activity against *Acanthamoeba quina,* and a greater than eight-fold increase of activity against *A. lugdunensis* in comparison to rosmarinic acid. Furthermore, we recorded significantly higher antimicrobial activity of the synthetized derivatives when compared to rosmarinic acid itself. Both synthetized quaternary phosphonium salts of rosmarinic acid appear to be promising antitumor and antimicrobial agents, as well as impressive molecules for further research.

## 1. Introduction

Rosmarinic acid (RA), as an ester of caffeic acid and 3,4-dihydroxyphenyllactic acid, is an important naturally-occurring phenolic secondary metabolite. It has been described as a tannin-like compound and was first isolated in 1958 by the Italian chemists Scarpati and Oriente from the plant *Rosmarinus officinalis*, after which it was named. The biogenesis of rosmarinic acid has been widely studied. The biosynthesis of RA begins with the amino acids l-phenylalanine and l-tyrosine. The caffeoyl part of the molecule is formed from phenylalanine through cinnamic and *p*-cumaric acids. The second part of the molecule, 3,4-dihydroxyphenyllactic acid, is formed from l-tyrosine through 4-hydroxyphenylpyruvic acid. RA is one of the most frequently occurring caffeic acid derivatives in the plant kingdom. It is a typical secondary metabolite for Lamiaceae plants, chiefly occurring in the Nepetoidae subfamily, which includes genera like *Mentha*, *Melissa*, *Salvia*, *Rosmarinus*, *Lycopus*, *Origanum*, *Thymus* etc. However, the presence of RA in the Lamiaceae outside of the Nepetoideae subfamily has also been reported in the genus *Teucrium*, *Aegiphila*, or *Hymenopyramis*. The occurrence of RA has been described in other families as well, e.g., Boraginaceae, Apiaceae, Scrophulariaceae, Rubiaceae, Rosaceae, Asteraceae, Araliaceae, and Cucurbitaceae [1,2,3]. RA exhibits a number of biological activities of which the most valuable are its antioxidant, anti-inflammatory and antimicrobial effects. Antibacterial activity has been proved against Gram-positive and even some Gram-negative bacteria [4,5,6,7]. Additional promising effects of RA are neuroprotection, cardioprotection and chemoprevention [8,9,10,11,12]. Research on RA during the last 10 years has been quite extensive. Many scientists have taken RA into their own *in vitro*, *in vivo*, or ex vivo experiments. RA is shown to stimulate liver regeneration [13,14]. Kantar-Gok et al. reported the potential benefits of RA in the prevention of auditory distortion that is related to estrogen deficiency and D-galactose administration. Rosmarinic acid reversed the AERP/MMN alterations in OVX D-galactose injected rats. Furthermore, RA may be efficient as an Alzheimer's disease treatment with its multiple bioactivities [15,16]. Orally administered rosmarinic acid may be excreted in the urine rather than in the bile, with a cleavage of ester bonds, selective *para*-dehydroxylation, methylation, and sulfate conjugation [17]. It is known that antioxidants such as vitamin E, ubiquinol or *N*-acetylcysteine bound to triphenylphosphonium cation (TPP) are selectively accumulated in mitochondria, and are more effective than underivatized molecules [18]. TPP contains positively charged phosphorus surrounded by large hydrophobic groups, allowing it to rapidly permeate lipid bilayers while retaining its positive charge. This positive charge facilitates accumulation in mitochondria due to differences in membrane potentials [19,20]. The biological activities of cationic amphiphilic compounds have been comprehensively summarized and it is known that they possess antibacterial, antifungal, antiprotozoal, antineoplastic, immunomodulatory effects and anti- Alzheimer action [21,22,23]. The hydrophilic properties of rosmarinic acid limit its penetration though the cell membrane and its intracellular action. The addition of lipophilic phosphonium cations to RA molecule may change its physical properties and affect its penetration though cell membranes. Therefore, the aim of this study was to prepare semi-synthetic amphiphilic derivatives of RA with positive effects on its biological activity.

## 2. Results and Discussion

### 2.1. Chemical Synthesis

**Scheme 1:**
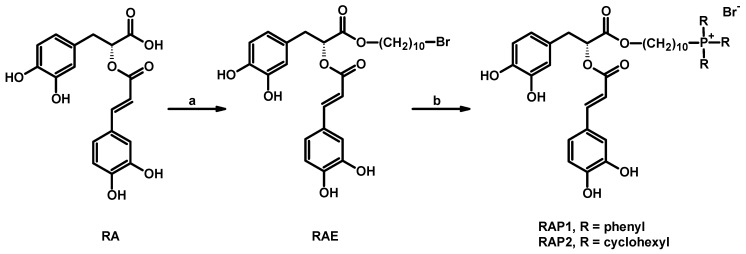
Preparation of quaternary phosphonium salts derived from rosmarinic acid. Reagents and Conditions: (**a**) 10-bromodecan-1-ol, triphenylphosphine, DIAD, THF, r.t. 48 h; (**b**) tertiary phosphine, acetonitrile, 80 °C, 48 h.

The synthesis of phosphonium salts derived from rosmarinic acid is depicted in Scheme 1. The modification of rosmarinic acid was performed in two steps. The first step was the esterification of rosmarinic acid with 10-bromodecanol. This was done by a Mitsunobu reaction performed according to a modified procedure [24]. Esterification was done in the presence of DIAD and triphenylphosphine. The ester **RAE** was prepared in moderate yield (62%). The next reaction step was the quarternisation. **RAE** was quarternised with either triphenylphosphine or tricyclohexyl-phosphine according to a modified previously published procedure [23]. The phosphonium salts **RAP1** and **RAP2** were obtained in yields of 9.2% and 52%, respectively. The poor yield of the, triphenylphosphonium salt **RAP1** was probably caused by lower reactivity of triphenylphosphine in comparison with tricyclohexylphosphine.

### 2.2. In Vitro Activity on A549, HCT116, HeLa, MCF-7 Cell Lines

The anticancer activity of RA and it´s mechanism of action was reviewed by Hossan et al., who described the antitumor effect of RA on cell lines of colorectal cancer, skin cancer, melanoma, lung cancer, oral cancer, leukemia, hepatoma, breast cancer and ovarian cancer [25]. Tao et al. determined the cell viability after exposing A549 cells (lung cancer) to 500 μM of various phenolcarboxylic acids and incubating them for 48 h. RA produced around 50% inhibition of cell viability with an IC_50_ value of 198.1 μM. The inhibitory effect on cell proliferation was attributed to COX-2 inhibition [26]. In the human breast cancer MCF7 cell line, RA reportedly inhibited DNA methyltransferase activity (88% inhibition at 40 μM RA) [27] and also has been found to dose-dependently inhibit the migration of MDA-MB-231BO human bone-homing breast cancer cells, which effect can prevent skeletal disorders found in breast cancer metastasis via modulating of NFκB ligand (RANKL)/RANK/osteoprotegerin, as well as by suppression of the expression of interleukin-8 [28]. In a recent study, the proliferation of colorectal cancer (CRC) cells (HCT116) was significantly inhibited by RA (50–200 μM, after 48 h) through the induction of cell cycle arrest and apoptosis. RA inhibits invasion and migration of CRC cells and decreases the expressions of matrix metalloproteinase (MMP)-2 and MMP-9. Furthermore, RA inhibited lung metastasis of CRC cells by activating AMPK in a mouse model [29]. Bacanli et al. studied the cytotoxic effect of RA on human epithelial adenocarcinoma (HeLa) cells, however, the IC_50_ values of RA could not be calculated at the concentrations they studied [30].

**Figure 1:**
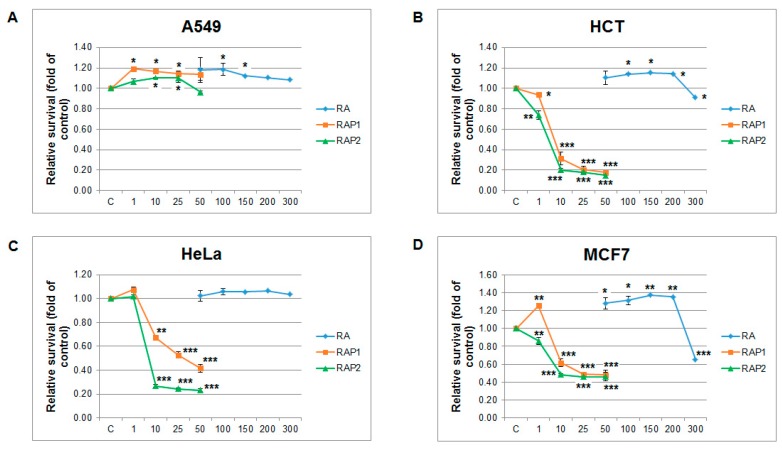
Effect of RA (c = 50–300 µM) and compounds **RAP1**, **RAP2** (c = 1–50 µM) on the metabolic activity of A549 (**A**), HCT116 (**B**), HeLa (**C**) and MCF-7 (**D**) cells using MTS assays. Data were obtained from three independent experiments, and significant differences were marked as * *p* < 0.05, ** *p* < 0.01, *** *p* < 0.001 versus control cells (untreated).

In our study, the antiproliferative effect of RA derivatives was determined by using the colorimetric MTS assay (Figure 1) on several cancer cell lines from different tissue origins (breast, colon, cervix and lung). The ideal IC_50_ values of the rosmarinic acid (RA) and newly synthesized derivatives of RA on human cancer cell lines are presented in Table 1.

Data are presented as the median of three independent experimental determinations performed in triplicate.

As the results show, the compounds **RAP1** and **RAP2** exhibited the most significant inhibitory effects on the growth of HCT116 cells (Figure 1), with IC_50_ values of 7.28 and 8.13 μM, respectively. Both tested compounds exhibited significantly stronger inhibitory effects on selected cancer cell lines than rosmarinic acid itself.

### 2.3. Antibacterial and Antifungal Properties

The antimicrobial activity of RA has already been proved on both Gram-positive and Gram-negative bacteria, as well as on yeasts [5,6,7]. According to the latest research, RA could be a candidate for a topical antimicrobial agent with killing activity on planktonic forms of clinical *S. aureus* strains, as well as with suppressive activity in the early stages of biofilm development [5]. Anticandidal effect was explained by inhibition of isocitrate lyase in glyoxylate cycle of *C. albicans* by RA with MIC 1000 mg/L [31]. In this study, the antimicrobial activity of RA and its newly synthesized derivatives was examined on seven bacterial collection strains (methicillin resistant *Staphylococcus aureus* CCM 4750, methicillin susceptible *S. aureus* CCM 4223, *Enterococcus faecalis* CCM 4224, *Pseudomonas aeruginosa* CCM 3955, *Klebsiella pneumoniae* CCM 4415, *Escherichia coli* CCM 3954, and *Proteus mirabilis* CCM 7188), one yeast (*Candida albicans* CCM 90028), and four mold collection strains (*Trichoderma viridae*, *Aspergillus flavus, Aspergillus niger*, and *Mucor racemosus*; all from the collection of Slovak Technical University in Bratislava, SK). A standardized broth microdilution assay was used for minimal inhibitory concentration (MIC) and minimal bactericidal concentration (MBC) testing [32,33]. In comparison to RA, both phosphonium salts (**RAP1**, as well as **RAP2**) exhibited a much stronger antibacterial effect (MBC 0.19–1.48 mM and 0.05–1.48 mM, respectively) on both Gram-positive and Gram-negative bacteria, and even a 144-times higher anti-candidal activity (MIC 0.012 μM in comparison to 1.73 μM of RA). **RAP2** was the most potent compound against the tested molds. However, the *Aspergillus flavus* strain was resistant to all samples within the range of evaluated concentrations (Table 2).

### 2.4. Anti-Acanthamoeba Activity

The antiprotozoal activity was studied against the free-living amoebae *Acanthamoeba spp.*, which are opportunistic protozoan parasites known as causative agents of granulomatous amoebic encephalitis (GAE), disseminated infections, and *Acanthamoeba* keratitis (AK) [34,35,36]. To date, no standard therapeutic procedures of *Acanthamoeba* infections have been developed, and searching for new potential amoebicidal agents continues. Although the antiprotozoal activity of RA was not studied, it is an important component of plant extracts exhibiting anti-*Acanthamoeba* activity, e.g., extracts from *Origanum* spp. [37] and *Teucrium* spp. [38]. In the present study, the antiprotozoal activity was investigated against two clinical isolates of T4 genotype: *Acanthamoeba lugdunensis* and *A. quina*. Both phosphonium salts exhibited considerably higher inhibitory activity than RA (Table 3). More than three-fold higher activity against *Acanthamoeba quina* and more than eight-fold higher activity against *A. lugdunensis* was detected in comparison with RA. Although the inhibitory activity of **RAP1** was similar against both isolates, the activity of **RAP2** was about four times higher against *A. lugdunensis* than against *A. quina*. However, from the perspective of practical use, the inhibitory activity of both phosphonium salts should be considered as moderate.

## 3. Materials and Methods

### 3.1. General Information

A rosmarinic acid standard was purchased by Sigma Aldrich (Sigma-Aldrich, St. Louis, MO, USA). All other chemicals used in the synthesis were obtained from commercial suppliers and were of p.a. purity. ^1^H-, ^13^C- and ^31^P-NMR spectra were measured on a MERCURY plus spectrometer (Varian, Palo Alto, CA, USA) working at frequencies of 300, 75, and 121.5 MHz, respectively. ^13^C- and ^31^P-NMR spectra were decoupled against protons. The spectra were measured in CDCl_3_ or CD_3_OD. The chemical shifts were referenced with respect to an internal TMS (δ^1^H = 0, δ^13^C = 0) or 85% H_3_PO_4_ (δ^31^P = 0 for ∈ ^31^P = 40.4807420 MHz) signal.

### 3.2. Synthesis of Compounds

#### 3.2.1. Synthesis of (1*R*)-2-(10-Bromodecyloxy)-1-(3,4-dihydroxybenzyl)-2-oxoethyl (2*E*)-3-(3,4-dihydroxyphenyl)acrylate (**RAE**)

Diisopropylazadicarboxylate (DIAD, 1.5 mmol; 320 µL) was added dropwise to a solution of 10-bromodecane-1-ol (1.5 mmol; 356 mg), rosmarinic acid (1.5 mmol; 541 mg) and triphenyl- phosphine (1.5 mmol; 420 mg) in anhydrous THF (5 mL). The reaction mixture was stirred at room temperature for 48 h, evaporated in vacuo, and purified by column chromatography over silica gel (CHCl_3_ → CHCl_3_/MeOH, 20/1, *v*/*v*). **RAE** was thus prepared in 62% yield: ^1^H-NMR (CDCl_3_, TMS) δ: 1.19–1.40 (m, 14H), 1.62–1.64 (m, 1H), 1.81–1.86 (m, 2H), 3.09–3.11 (m, 2H), 3.37 (t, *J* = 6.9 Hz, 2H), 4,13 (t, *J* = 6.6 Hz, 2H), 5.30 (m, 1H), 6.09 (d, *J* = 15.9 Hz, 1H), 6.65 (dd, *J* = 2.1 Hz, *J* = 8.1 Hz, 1H), 6.72 (d, *J* = 1.8 Hz, 1H), 6.75 (d, *J* = 8.1 Hz, 1H), 6.89 (dd, *J* = 8.1 Hz, *J* = 1.8 Hz, 1H), 7.00 (d, *J* = 1.8 Hz, 1H), 7.41 (d, *J* = 15.9 Hz, 1H); ^13^C-NMR (CDCl_3_, TMS) δ: 25.7, 28.1, 28.3, 28.7, 29.1, 29.3, 29.7, 32.8, 34.1, 36.7, 66.5, 73.3, 76.6, 77.0, 77.2, 77.5, 113.1, 114.5, 115.6, 116.9, 121.7, 126.7, 128.2, 143.0, 143.6, 143.7, 146.8, 167.8, 171.5.

#### 3.2.2. Synthesis of the Phosphonium Salts (10-[((2*R*)-3-(3,4-Dihydroxyphenyl)-2-{[(2*E*)-3-(3,4- dihydroxyphenyl)prop-2-enoyl]oxy}propanoyl)oxy]-P,P,P-triphenyldecane-1-phosphonium bromide (**RAP1**) and (*P*,*P*,*P*-tricyclohexyl-10-[((2*R*)-3-(3,4-dihydroxyphenyl)-2-{[(2*E*)-3-(3,4- dihydroxyphenyl) prop-2-enoyl]oxy}propanoyl)oxy]decane-1-phosphanium bromide (**RAP2**)

Tertiary phosphine (triphenylphosphine: 0.45 mmol; 118 mg or tricyclohexylphosphine 0.587 mmol; 165 mg) and **RAE** (0.345 mmol; 200 mg resp. 0.587 mmol; 340 mg) were dissolved in acetonitrile (5 mL). The mixture was stirred at 81 °C for 48 h, cooled down to room temperature and acetonitrile was evaporated under reduced pressure. Products were purified by column chromatography over silica gel (CHCl_3_ → CHCl_3_/MeOH, 4/1, *v*/*v*) to give **RAP1** (9.2%) and **RAP2** (52%) as yellowish solids. **RAP1:** ^1^H-NMR (CD_3_OD, TMS) δ: 1.17–1.38 (m, 10H), 1.22–1.75 (m, 6H), 3.02 (d, *J* = 6.6 Hz, 2H), 3.22–3.38 (m, 2H), 4.07 (t, *J* = 6.1 Hz, 2H), 5.14 (t, *J* = 6.3 Hz, 1H), 6.21 (d, *J* = 15.9 Hz, 1H), 6.56 (dd, *J* = 8.1 Hz, *J* = 1.8 Hz, 1H), 6.69 (d, *J* = 8.1 Hz, 1H), 6.72 (d, *J* = 1.8 Hz, 1H), 6.75 (d, *J* = 8.1 Hz, 1H), 6.89 (dd, *J* = 8.1 Hz, *J* = 1.8 Hz, 1H), 7.00 (d, *J* = 1.8 Hz, 1H), 7.51 (d, *J* = 15.9 Hz, 1H), 7.70–7.91 (m, 15H); ^13^C-NMR (CD_3_OD, TMS) δ: 21.3 (d, *J* = 50.6 Hz), 22.1 (d, *J* = 4.3 Hz), 25.4, 28.1, 28.4, 28.6, 28.7, 28.9, 30.1 (d, *J* = 16.1 Hz), 36.5, 65.0, 73.5, 112.8, 113.9, 114.9, 115.2, 116.3, 118.5 (d, *J* = 85.7 Hz), 120.4, 121.9, 126.0, 127.3, 130.1 (d, *J* = 12.6 Hz), 133.3 (d, *J* = 9.9 Hz), 134.9 (d, *J* = 2.9 Hz), 144.0, 144.9, 145.6, 146.5, 148.6, 167.0, 170.4; ^31^P-NMR (CD_3_OD, TMS) δ: 23.7; **RAP2:** ^1^H-NMR (CD_3_OD, TMS) δ: 0.78–2.60 (m, 51H), 3.04 (d, *J* = 6.6 Hz, 2H), 3.22–3.38 (m, 2H), 4.10 (t, *J* = 6.1 Hz, 2H), 5.16 (t, *J* = 6.3 Hz, 1H), 6.27 (d, *J* = 15.9 Hz, 1H), 6.58 (dd, *J* = 8.1 Hz, *J* = 1.8 Hz, 1H), 6.69 (d, *J* = 8.1 Hz, 1H), 6.70 (d, *J* = 1.8 Hz, 1H), 6.79 (d, *J* = 8.1 Hz, 1H), 6.97 (dd, *J* = 8.1 Hz, *J* = 1.8 Hz, 1H), 7.05 (d, *J* = 1.8 Hz, 1H), 7.56 (d, *J* = 15.9 Hz, 1H); ^13^C-NMR (CD_3_OD, TMS) δ: 21.9 (d, *J* = 4.3 Hz), 25.1, 25.4, 26.1 (d, *J* = 11.9 Hz), 26.6 (d, *J* = 3.8 Hz), 28.1, 28.3, 28.6, 28.8, 28.9, 29.4 (d, *J* = 40.7 Hz) 30.6 (d, *J* = 16.1 Hz), 36.5, 64.9, 73.6, 112.8, 113.9, 114.9, 115.2, 116.1, 120.4, 121.8, 126.1, 127.3, 144.1, 144.9, 145.5, 146.5, 148.6, 167.0, 170.4; ^31^P-NMR (CD_3_OD, TMS) δ: 31.8.

### 3.3. Anticancer Activity In Vitro

The compounds were dissolved in dimethyl sulfoxide (DMSO, Sigma-Aldrich, St. Louis, MO, USA). The final concentration of DMSO in the culture medium was <0.2% and exhibited no cytotoxicity. The human cancer cell line HCT116 (human colorectal adenocarcinoma) and HeLa (human cervical adenocarcinoma) was cultured in RPMI 1640 medium (Biosera, Kansas City, MO, USA). MCF-7 (human breast adenocarcinoma) and A549 (human lung adenocarcinoma) cell lines were maintained in a growth medium consisting of high glucose Dulbecco’s Modified Eagle Medium with sodium pyruvate (GE Healthcare, Piscataway, NJ, USA). The growth medium was supplemented with a 10% fetal bovine serum, penicillin (100 IU/mL) and streptomycin (100 μg/mL) (all Invitrogen, Carlsbad, CA, USA) in an atmosphere containing 5% CO_2_ in humidified air at 37 °C. Cell viability, estimated by trypan exclusion, was greater than 95% before each experiment.

MTS cell proliferation/viability assay: The metabolic activity colorimetric assay (MTS) was used to determine the effects of RA (c = 50–300 µM) and compounds **RAP1**, **RAP2** (c = 1–50 µM) on the metabolic activity of several cell lines. After 72 h of incubation, 10 µL of MTS (Promega, Madison, WI, USA) was added to each well according to the CellTiter 96^®^ AQueous One Solution Cell Proliferation Assay protocol. After minimum 1 h incubation, the absorbance was measured at 490 nm using the automated CytationTM 3 Cell Imaging Multi-Mode Reader (Biotek, Winooski, VT, USA). The absorbance of the control wells was taken as 1.0 (100%) and the results were expressed as a fold of the control. All experiments were performed in triplicate. Ideal IC50 values were calculated from MTS analyses.

### 3.4. Antibacterial Activity Testing

The minimal inhibitory (MIC) and minimal bactericidal concentrations (MBC) of the tested compounds were detected by broth microdilution assay according to the EUCAST recommendations [32]. The tested compounds were dissolved in 50% ethanol, diluted 1:1 in double strength Mueller-Hinton broth (OXOID, Basingstoke, UK) and afterwards, serial two-fold dilutions were prepared. The tested concentrations of RA, **RAP1** and **RAP2** ranged from 13,877 to 14 μM, from 5940 to 6 μM and from 5815 to 6 μM, respectively. One hundred μL aliquots of the tested substances in particular dilutions were placed to the U-shaped sterile microtiter plate wells. Bacterial inocula were prepared in Mueller-Hinton broth from the overnight bacterial cultures on blood agar. They were adjusted to 5 × 10^6^ CFU/mL and inoculated in 10 μL aliquots into the wells containing the tested compounds. Antimicrobials-free Mueller-Hinton broth containing only the diluent in two-fold serial dilutions was used for bacterial growth control, and wells containing broth and serial dilutions of the tested compounds, yet free of bacterial inoculum, were included as both the sterility and negative growth controls. MIC was detected after an overnight cultivation at 35 °C in ambient air, as the lowest concentration of the antimicrobial compound inhibiting the growth of bacteria. MBC was determined after dot-inoculation of 3 μL aliquots from wells with inhibited bacterial growth on antimicrobial-free agar medium plates. The plates were cultivated overnight at 35 °C and the MBCs were determined as the lowest concentrations of the tested compounds able to inactivate 99.9% of the tested bacterial inocula.

### 3.5. Antifungal Activity Testing

*In vitro* antifungal susceptibility testing of *Aspergillus flavus*, *Aspergillus niger*, *Mucor racemosus*, and *Tirchoderma viridae* strains was determined by a broth microdilution technique following the guidelines of the EUCAST [33] for conidia forming molds. RPMI 1640 with L-glutamine and pH indicator bicarbonate supplemented with glucose to a final concentration of 2% and MOPS to final a concentration 0.165 M, pH 7.00 was used for susceptibility testing. The tested agents as a powder were diluted in DMSO as a stock solution with a concertation of 10 mM. The stock solution was used for preparing working solutions in a cultivation medium (1 mM). Serial doubling dilutions of samples were prepared in RPMI medium. All mold species were cultivated on Potato Dextrose Agar (PDA) plates for 5 to 7 days at 25 °C. Stock spore suspensions were prepared by washing the surface of the plates with sterile saline containing 0.01% Tween 80. Spore suspensions were counted with a haemocytometer and diluted in sterile distilled water to a concentration of 2 − 5 × 10^5^ CFU/mL. Wells were inoculated with 100 µL of spore suspension (final concentration 1 − 2.5 × 10^5^ CFU/mL). As a positive control, 100 µL of RPMI 2% glucose was dispensed into a well and inoculated, and RPMI 2% glucose was also inoculated. As a negative control, 100 µL of RPMI 2% glucose was dispensed into a well and 100 µL of RPMI 2% glucose with 100 µL of distilled water used for dilution of spore suspension. Microdilution plates were incubated without agitation at 30 °C for 48 h. The results were read visually by light microscopy (magnification 40×). MIC was determined as a concentration of drug yielding no conidia forming. Susceptibility testing was performed in duplicate in two independent experiments. Activity of the tested agents against *Candida albicans* strain was performed according to the EUCAST-recommended method for the determination of broth dilution minimum inhibitory concentrations of antifungal agents for yeasts with some modifications. The yeast inoculum was prepared in Sabouraud broth from 48-h culture of the tested strain grown on Sabouraud agar. The inoculum was adjusted to a concentration of 0.5 × 10^6^ CFU/mL. RA, and its tricyclohexylphosphine salts solutions were prepared similarly according to the protocol used for bacteria and diluted in Sabouraud broth. The wells with tested agents were inoculated by 10 μL aliquots of yeast suspension and cultivated for 24 h at 35 °C in ambient air. Similar control wells were included like in the protocol of antibacterial activity testing. The MIC was evaluated as the lowest concentration inhibiting the growth of the tested yeasts.

### 3.6. In Vitro Amoebicidal Activity Assay

The compounds were dissolved in dimethyl sulfoxide (DMSO, Sigma-Aldrich, St. Louis, MO, USA). The highest final concentration of DMSO in water used to dissolve the compounds was 1% and exhibited no cytotoxicity. The cytotoxic activity against two clinical isolates of free-living amoebae, *Acanthamoeba lugdunensis* (strain AcaVNAK02, T4 genotype) and *A. quina* (strain AcaVNAK03, T4 genotype), isolated from the corneas of two patients with *Acanthamoeba* keratitis, was tested in vitro as previously described [39]. In brief, from the 2-day monoxenic cultures on agar plates, the trophozoites were axenized by inoculation into the Bacto-Casitone/Serum medium (BCS) with penicillin and ampicillin. After 72 h, the active trophozoites were transferred into peptone-yeast extract-glucose medium (PYG) with penicillin and ampicillin. After 5 passages, the trophozoites were transferred into the PYG medium without antibiotics and subsequently cultivated in this medium. Cytotoxicity measurements were carried out in 96-well microtiter plates under sterile conditions at 37 °C. Each well was seeded with 100 µL (2 × 10^5^ cells ml^-1^) of a trophozoite suspension. Afterwards, 100 µL of a freshly prepared medium containing a tested compound at 6 concentrations was added to all wells, except for the untreated control wells that received 100 µL of pure medium. Each compound was tested at final concentrations of 500, 250, 125, 62.5, 31.25, and 15.6 µM. The reduction of trophozoites was recorded after 24 h by counting the surviving cells in a Bürker-Türk hemocytometer. Viability of trophozoites was determined by trypan blue exclusion; 100% eradication was confirmed by transferring 50 µL of the suspension to a PYG medium and subsequently amoeba growth was recorded for 14 days. The EC50 (effective concentration of tested compound that reduces the survival of amoebae by 50%) values were calculated by linear regression analysis using Microsoft Office Excel 2010 (Microsoft Corporation, Redmond, WA, USA). All experiments were performed in quadruplicate for each concentration.

## 4. Conclusions

Two quaternary phosphonium salts were prepared from rosmarinic acid and tested for their inhibitory effect on selected cancer cell lines and for their antimicrobial and antiprotozoal properties. In summary, both the triphenylphosphine salt of RA (**RAP1**) and tricyclohexylphosphine salt of RA (**RAP2**) exhibit strong inhibitory effects on cancer cell lines, moderate anti-*Acanthamoeba* activity, strong antibacterial and anti-candidal, as well as moderate anti-mold effects in comparison to RA, and appear to be perspective antitumor and antimicrobial agents. In general, **RAP2** possesses higher activities in comparison with **RAP1**. The tricyclohexylphosphonium cation is more lipophilic in comparison with the triphenylphosphonium one, so the higher lipophilicity of **RAP2** probably improved penetration of rosmarinic acid though the cell membrane and increased its biological effects in all tests.

## Figures and Tables

**Table 1:** The IC_50_ (µM) values of RA and its phosphonium salts **RAP1** and **RAP2** in different cell lines after 72 h incubation.

Compound	Cancer Cell Lines			
	**A549**	**HCT116**	**HeLa**	**MCF-7**
RA	>300	>300	>300	>300
**RAP1**	>300	7.28	32.67	19.36
**RAP2**	132.25	8.13	7.20	16.54

**Table 2:** Antibacterial and antifungal activity of RA and its phosphonium salts **RAP1** and **RAP2.**

	**Antibacterial Activity (MIC and MBC Values, mM)**																
	***S. aureus*** **CCM 4750 ^1^**		***S. aureus* CCM 4223 ^2^**			***E. faecalis* CCM 4224**			***P. aeruginosa* CCM 3955**		***K.pneumoniae*** **CCM 4415**			***E. coli* CCM 3954**		***P. mirabilis* CCM 7188**	
	**MIC**	**MBC**	**MIC**		**MBC**	**MIC**	**MBC**		**MIC**	**MBC**	**MIC**		**MIC**	**MIC**	**MBC**	**MIC**	**MBC**
RA	3.47	3.47	3.47		3.47	3.47	13.88		3.47	3.47	3.47		3.47	1.73	3.47	3.47	3.47
**RAP1**	0.19	0.19	0.19		0.19	0.19	0.74		0.37	0.74	0.74		0.74	0.37	0.74	0.74	1.48
**RAP2**	0.05	0.05	0.05		0.05	0.05	0.18		0.36	0.73	0.36		0.36	0.18	1.45	0.36	0.73
	**Antifungal activity (MIC values, mM)**																
	***Trichoderma viridae***			***Aspergillus flavus***				***Aspergillus niger***				***Mucor racemosus***			***Candida albicans*** **CCM 90028**		
RA	0.5			>0.5				>0.5				0.5			1.73		
**RAP1**	0.25			>0.5				0.5				0.25			0.012		
**RAP2**	0.062			>0.5				0.5				0.125			0.012		

^1^ MRSA—methicillin resistant *S. aureus*; ^2^ MSSA—methicillin susceptible *S. aureus*; MIC—minimal inhibitory concentration; MBC—minimal bactericidal concentration; CCM—Czech Collection of Microorganisms.

**Table 3:** The values of EC50 (mM) of RA and its phosphonium salts **RAP1** and **RAP2** on *Acanthamoeba* trophozoites after 24 h of incubation.

Compound	*Acanthamoeba lugdunensis* (AcaVNAK02)	*Acanthamoeba quina* (AcaVNAK03)
RA	11.3 ± 0.2	19.1 ± 0.4
**RAP1**	1.24 ± 0.03	1.14 ± 0.05
**RAP2**	1.32 ± 0.08	5.6 ± 0.3

Data are presented as mean values ± standard deviation of four independent experiments.
